# Use of Social Media for Health Advocacy for Digital Communities: Descriptive Study

**DOI:** 10.2196/51752

**Published:** 2023-11-14

**Authors:** Chidimma Ogechukwu Ezeilo, Nicholas Leon, Anushka Jajodia, Hae-Ra Han

**Affiliations:** 1 Johns Hopkins University Bloomberg School of Public Health Baltimore, MD United States; 2 Johns Hopkins University School of Nursing Baltimore, MD United States

**Keywords:** social media, health advocacy, community health, Twitter, health communication, health promotion, communication, communications, advocacy, tweet, tweets, nurse, nurses, nursing

## Abstract

**Background:**

There has been a growth surge in the use of social media among individuals today. The widespread adoption of these platforms, coupled with their engaging features, presents a unique opportunity for the dissemination of health advocacy information. Social media is known as a powerful tool used to share health policy and advocacy efforts and disseminate health information to digital community members and networks. Yet, there is still a gap in the full exploitation of this powerful instrument, among health care professionals, for health advocacy campaigns.

**Objective:**

This paper aims to describe the process of mobilizing social media platforms such as Twitter (rebranded to X Corp in 2023) for health advocacy of the digital community. Additionally, it aims to share the lessons and insights gained during this digital health advocacy engagement process.

**Methods:**

We performed a comprehensive review of Twitter analytical data to examine the impact of our social media posts. We then consolidated these analytic reports with our meeting logs to describe our systematic, iterative, and collaborative design process to implement social media efforts and generate key lessons learned.

**Results:**

Our review of monthly Twitter analytical reports and regular team meeting logs revealed several themes for successful and less successful practices in relation to our social media–based health advocacy efforts. The successful practices noted by the team included using personable, picture-based tweets; using a series of posts on a particular topic rather than an isolated post; leveraging team members’ and partners’ collaborations in shared posts; incorporating hashtags in tweets; using a balanced mix of texts and graphics in posts; using inclusive (nondestigmatizing) languages in tweeted posts; and use of polls to share tweets. Among the many lessons learned, we also experienced limitations including a lack of comprehensive statistics on Twitter usage for health care–related purposes such as health advocacy and limits in collating the estimates of the actual impact made on the intended digital community members by our posts.

**Conclusions:**

Twitter has been successfully used in promoting health advocacy content, and the social media team aims to explore other social media platforms that have a wider reach than Twitter. We will continue making necessary adjustments in strategies, techniques, and styles to engage the audience as we expand onto new platforms like Instagram and TikTok for health advocacy promotions.

## Introduction

Social media refers to any form of electronic communication that allows users to create and share content through web-based communities [1], hence facilitating engagement between individuals. According to the Pew Research Center [2], 73% of adults in the United States use at least one form of social media. The use of social media is even higher among health professionals. One study found 88% of health care workers surveyed used at least one form of social media [3]. Most social media users are younger than 50 years across nearly all social media platforms [2,3].

Social media’s widespread use across the United States makes it an important and powerful tool today as technology is rampantly accessible for almost anyone via mobile phones, PCs, etc. Indeed, social media has been used as an effective means to engage stakeholders, participate in health policy and advocacy efforts, and disseminate health information to community members [4]. In the health care space, social media offers the opportunity to modify the health behaviors of individuals, reaching inaccessible or isolated audiences across wide geographic locations with just a click [5,6]. The strategic use of the key features of social media such as hashtags (which is any word or phrase immediately preceded by the # symbol), tagging, or retweeting may have an impact on the way social media is received by the intending audience.

The Center for Community Programs, Innovation and Scholarship (COMPASS) at the Johns Hopkins School of Nursing is an operational unit for the school’s community-based health education and wellness services (eg, blood pressure screening and flu vaccination) with the goal of promoting the health of individuals with limited resources in the neighborhoods [7]. Since its inception in 1996, COMPASS has emphasized health equity through service learning for our faculty and students and health advocacy for our communities. Health advocacy particularly encompasses diverse activities that promote “health and access to health care in communities and the larger public” [8].

With the increasing popularity and uptake of social media, COMPASS faculty, staff, and students decided to mobilize social media platforms for health advocacy to reach out to the digital community. Despite social media’s widespread use, it remains an underused tool by health professionals such as nurses to share health information and connect with organizations, stakeholders, and other nursing professionals [9]. COMPASS chose to use Twitter (recently rebranded to X Corp) to bridge this communication gap due to its demonstrated low cost and potential high reach and impact with community members [10]. The affordances of Twitter including the use of post text, pictures, and tweets of about 160 characters, with its ability to connect with audiences through liking a post, and retweeting someone else’s tweet or post, also made Twitter a very attractive platform for the team to promote health-related messages and information. Twitter is the most prominent microblogging site, a web service that allows subscribers to send short messages to other subscribers, with over 140 reported health care uses including community outreach and health advocacy [11]. To this end, a social media team (including a mix of professionals with or without nursing background) out of COMPASS membership was created to plan and implement regular social media–based health advocacy postings.

Currently, the social media team involves 2 graduate students in schools of nursing and public health and a design strategist. Together, they work closely with COMPASS faculty and staff. During their weekly or biweekly meetings, the social media team (also referred to as the “team” hereafter) identifies 1-3 monthly topics to address on social media and follows a series of steps to post targeted messages on the platform. Posted messages are reviewed with detailed analytics of the previously shared content during the center’s monthly meeting for progress updates and feedback to the team. The purposes of this paper are two-fold: (1) to describe our process in generating, crafting, and posting target messages and (2) to share lessons learned so far in using social media for health advocacy in communities in the digital space, where physical interaction is gotten.

## Methods

### Brainstorming or Planning

The social media team conducted initial research on how other centers and organizations promote health advocacy as sources of inspiration (eg, World Health Organization, Prison Policy Initiative, and Center for Public Health Advocacy). The team reviewed the available documents (including posts, videos, comments, images, and web links) that these organizations had previously shared on Twitter. In this review, the team studied the structure of the tweets made and the reaction from the web-based audience. The tweet structures considered included the use of texts, data or numbers, and smileys or emoticons in tweets posted. The initial review of these documents allowed the team to develop an extensive outlook of the different methods, which had worked for organizations, in promoting health advocacy topics on social media platforms.

Next, the team inquired and clarified with COMPASS faculty on what health topics COMPASS would advocate for while also discussing which potential audiences to target such as the school’s faculty and students, health practitioners, advocates, policy makers, and community members. The team was guided to ensure that topic selections and key messages were closely aligned with COMPASS’ main goals centered around neighborhood-based wellness, health promotion, social determinants of health, and health equity.

The social media team looked at various message formats for health advocacy: policy briefs, data visualizations, art or infographics, and fact sheets. Thereafter, the team settled to focus on one of the following types of templates to facilitate ease of reading and better reach to a wider audience: (1) data visualizations, infographics, photographs, art, GIF-focused posts to highlight statistics, facts, quotes, stories, and behind-the-scenes or “in-the-field” work at the center and (2) web-based polls such as questions and answers on various topics to engage in. The social media team also decided to conduct monthly collation of target health topics followed by weekly team meetings to generate graphics and messages, and finally, tweeting regularly with monthly analytics presented to the whole team.

### Implementation

Our design process for each monthly set of Twitter posts begins with task division among team members. These tasks include (1) gathering appropriate research data; (2) simplifying content using plain language and fewer words; (3) designing an attractive, engaging, and easy-to-read post; (4) crafting the Twitter caption; and (5) posting on Twitter. We use the web-based design software popularly referred to as “Canva” (Canva Pty Ltd) to create our posts as the software offers numerous design templates and options for team members to collaborate on designs. This process repeats every month.

Once a month, the social media team generates a monthly Twitter Analytics report and shares it with the members of COMPASS. The monthly analytics report includes detailed information about tweets such as number of tweets posted, number of COMPASS profile visitations, number of new followers, and tweet impressions, mentions, and engagement rate (ie, the number of engagements divided by the number of impressions). Using these analytics, a feedback meeting is held to analyze the successes and points of possible improvements on the previous month’s posts and to discuss necessitated change tactics in the style of posting. This regular evaluation is helpful as the team uses the opportunity to tweak the method of tweeting, majorly sticking to styles that had significant engagement rates in the past month and exploring new methods of social involvement with the community on Twitter. Team collaboration is essential to create impactful posts in a short time and flexible manner.

Table 1 provides a snapshot of the analytical data for the past 1 year used by the team to inform decisions of the next posts on the platform. The regular shifts in the number of tweets, profile visits, new followership, tweet impressions, and mentions are important details that influence the team monthly when considering relevant content to share. From Table 1, the team shared about 9 posts monthly with profile visits of over 700. The COMPASS page attracted approximately 7 new followers monthly and got mentioned by other Twitter users about 7 times monthly.

**Table 1:** Monthly tweet analytics for April 2022 to March 2023.

	2022										2023				Monthly average
Month	Apr	May	Jun	Jul	Aug	Sep	Oct	Nov	Dec	Jan		Feb	Mar		
# tweets, n	10	7	6	8	10	6	10	9	15	8		13	10	9	
# profile visits, n^a^	782	580	804	1104	1286	615	1301	564	866	—^b^		598	790	774	
# new followers, n^c^	8	10	8	5	5	6	8	7	9	6		6	9	7	
Tweet impressions, n^d^	3713	4076	3073	2785	3995	4144	5573	4245	4493	3375		7822	4789	4340	
Mentions, n^e^	9	5	1	—^b^	14	2	14	20	3	-		8	8	7	

^a^Number of times users visited the Twitter profile page.

^b^Not available.

^c^Number of new followers the Twitter account has gained.

^d^The total number of times Twitter users saw the tweet.

^e^Tweets in which our Twitter username was included by the software Canva.

### Ethical Considerations

The Johns Hopkins Medicine Institutional Review Board has determined that the study qualifies as exempt research under the Department of Health and Human Services regulations.

## Results

### Key Findings

Our review of monthly Twitter Analytics reports along with regular team meeting logs revealed several themes for successful and less successful practices in relation to our social media–based health advocacy efforts. Several key lessons learned are highlighted in the following paragraphs.

### Personable, Picture-Based Tweets to Promote User Engagement

The social media team used Twitter as a platform to showcase the research and advocacy work of COMPASS members, promotions for regular scholarly events led by COMPASS (eg, monthly journal club discussions focusing on articles addressing health equity and social determinants of health), and picture-based promotions of community partnered health and wellness events. For example, some of the posts showcased several COMPASS members at a community health fair providing free blood pressure screenings and educational materials to community members and distributing resources in the community. We found that the personable, picture-based approach generated some of the highest rates of engagement.

### Single Versus a Series of Posts

The social media team tested 2 different styles of posting tweets to promote health topics related to the work and mission of COMPASS. The first style was to post just once. For example, the team focused on sharing relevant information via 1 Twitter post on the “maternal mortality rate” in Baltimore city or in the state of Maryland as a whole. The post received 2 retweets and 4 likes. Likes are achieved by tapping the heart icon to like a tweet, and the author will see that you appreciate it. The second style was to share more than 1 post in a consecutive manner. For example, the social media team made a series of posts focusing on “busting myths about diabetes.” The posts received 6 retweets and 10 likes. The team noticed increased engagement and decided to expand and strengthen the strategy of posts by moving away from just single isolated posts toward a “series approach” of sharing several posts on the same health topic. With this renewed approach, the social media team posted a series of 5 Twitter posts focusing on “health literacy.” The posts received 43 retweets and 70 likes. The increase in engagement might have been due to an iterative posting strategy that we were able to tailor based on the observed change in reactions and engagement of the Twitter community.

### Leveraging Team Members and Partners

Tweets posted with images or pictures showing collaboration between the COMPASS team and community members received the highest level of Twitter engagement (such as retweeting, sharing, and commenting) as compared to other Twitter posts shared without any visual image of the team’s partnerships with community members. This may be due in part to COMPASS members and community partners having an incentive to promote content that shows pictures of themselves, their work, and their colleagues. The ability to directly tag members’ and partners’ accounts on Twitter sends them a notification of our post, thus encouraging them to “like” and “retweet” our content. When member and partner accounts “like” and “retweet” our posts, our content is then shared among their Twitter followers. Indeed, the Twitter accounts of community organizations we have worked with are tagged on COMPASS community partnership–focused posts to encourage engagement on our posts, which in turn increased the total number of Twitter users able to view COMPASS’ work in the community. An example tweet that tagged COMPASS partners at a health education fair received an engagement rate of 12.2%. Engagement is defined as the total number of times users interacted with a tweet such as clicking on hashtags and links, posting account’s profile avatar, username, and tweet expansion, or liking, retweeting, replying, and following the tweet or account [12]. For health advocacy–related content to reach the largest possible audience, we found that it is helpful to include related members in posts through tagging.

### Using Hashtags

A hashtag is any word or phrase immediately preceded by the # symbol. When one clicks or taps on a hashtag, other tweets containing the same hashtags appear. Hashtags are essential elements when posting on social media. The rate of using hashtags influences the rate of engagement on each post for health advocacy promotion. Sample hashtags our team used, *#cervicalcancerelimination* or *#globalcancer*, amassed over 200 more impressions than posts without hashtags. This may be by virtue of the interactive function of hashtags. When using a hashtag in a tweet, “it becomes linked to all the other tweets that include it” [13]. This “linking” structure through hashtags allows people to easily follow topics and themes of interest, hence increasing the reach of our tweet posts.

### Using Numbers or Words Only Versus Both Elements

**Figure 1:**
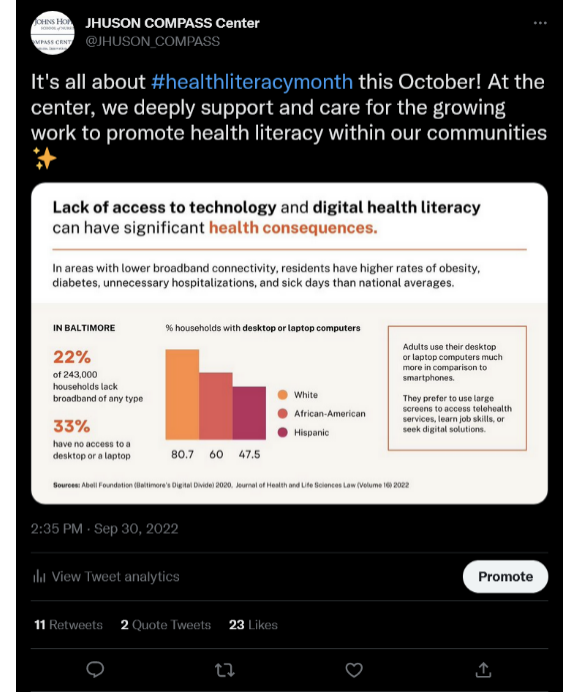
A tweet example showing a mixed use of texts and numbers.

A balanced combination of both words and numbers use on each posted tweet is critical in generating the most views and engagement from the Twitter community. Our experience indicates that posts containing only words or numbers received less engagement compared to posts containing a combination of both elements. For example, we created a post describing the importance of using plain language in health communications using only words, and the post received 7 retweets and 10 likes. Another post that contained predominantly numerical information on health literacy–related expenditures received a similar 7 retweets and 11 likes. In contrast, a post containing statistics on digital health literacy used a balance of words and numbers, and the post received 10 retweets and 14 likes (Figure 1). This demonstrates that a good combination of numbers and words are important to make a post more engaging on Twitter.

### Proper Framing of Language for Health Advocacy

Language matters, and information shared may be perceived incorrectly if the administrator of the Twitter post is unaware of the “right” language to use for the community. One initial challenge for us was knowing how to frame posts made for health advocacy for community audiences. The team used person-first terms and destigmatizing language to ensure there was no use of demeaning words or references toward targeted audiences. Examples of such terms included using “those living with hypertension” rather than “hypertensive patients” or “those living with diabetes” rather than “diabetic patients.” The use of a meticulous language structure, which was continually changed as posts were made, was further improved after the team partook in training opportunities such as social media conferences or simply expanding team members’ language knowledge base by researching and referring to available resources. Additionally, the team was mindful of not taking the role of primary care providers to provide medical advice via posts. We chose to offer evidence-based information and data so people can advocate for issues they care about or take questions to discuss with health care providers to make informed decisions.

### Use of Polls to Share Tweets

The research team also used polls and stimulating questions as mechanisms to enhance community involvement with shared tweets. This participatory model served as a gauge for the community’s receptiveness to our content. Posts tagged with “Did you know?” were expected to elicit some reaction among the community members. Although the intention was to encourage the community to use the comment section to share their thoughts on the polls or questions asked, the level of engagement did not reach the team’s anticipated levels. In view of this, future posts will explore the regular use of the feature “Twitter chats” as a strategy to encourage participation from community members and foster conversations in the realm of health advocacy. The implementation of Twitter chats may help expand our network and heighten expectations for engagement with questions and polls.

## Discussion

### Insights

The use of social media is growing, and Twitter (or X) continues to be a popular communication channel to post information and exchange content. The COMPASS team has used Twitter regularly and successfully as a tool to promote health advocacy. We found that social media posts are using pictures with a series of themed messages, and leveraging partners yielded some of the highest engagement rates so far.

Engagement rate on Twitter is important because it is a reliable indicator of the tweet’s performance and shows the percentage of interaction the Twitter community had with the content posted. The definition of “high” or “low” engagement rates on Twitter may vary, but generally, an engagement rate ranging from 0.09% to 0.33% is considered high [14]. Our picture-based, series of posts on health literacy had an engagement rate of 12.3%, showing the great impact pictures had on the spread of our tweets. Our experience is similar to what has been reported in the literature. For example, in a study of young adults to explore their perspectives on strategies to promote vegetable intake, social media posts using pictures were ranked most motivating, as opposed to awareness-raising posts that received a lower ranking [15]. When using pictures, our team focused on infusing culturally sensitive and inclusive visuals to represent diverse communities and topics across Twitter posts. A recent qualitative study of mommy bloggers also revealed that diverse images helped them relate better to the information and made them feel represented [16]. Considering the rise in the use of social media as a health communication vehicle, it would be important to use visual images to attract and hold the attention of all types of people [17].

Leveraging community and professional partners helped us increase engagement and viewership of our health advocacy messages on social media. Leveraging partners has been an underused tactic in social media advocacy. An analysis of 750 randomly selected tweets from 188 different civil rights and advocacy organizations found that only 2% of sampled tweets leveraged partners through showcasing public events and direct action [18]. In the analysis, only 0.27% (ie, 3 of 750 Tweets analyzed) of sampled tweets incorporated the advocacy tactic of coalition building by promoting community and professional partnerships. Despite underuse, our experience shows that leveraging partnerships through social media can be one of the most successful ways of promoting engagement on social media.

One of the features on Twitter, that is, “hashtag,” connects users to varied topics that they can follow based on interest under an umbrella designated by a name, irrespective of who is making the post [19]. Research has shown the value of hashtags for promoting diverse health-related themes or events such as conferences, chats, and journal clubs [20-23]. A recent analysis of tweets containing the hashtag #PsychTwitter from 2019 to 2022 shows the massive impact of a hashtag when it is used on a global scale [21]; the #PsychTwitter resulted in 125,297 tweets and 492,565,230 impressions, with the United States being the top country with the most users. While most published studies on hashtags have explored the role of social media as a platform for providing support and information about varying health topics, there is limited research addressing engagement performance (eg, number of impressions or tweets) with certain hashtags. Future research is warranted to compare the health advocacy outreach achieved by tweets with and without hashtags.

One of our key lessons learned was the importance of adequate framing of messages using plain language for health advocacy on Twitter. Given the nature of Twitter as a microblogging (short blog post for quick and direct interactions) service, health providers, researchers, and lay public can comment on diverse health topics. Through a qualitative study examining 665 tweets [24], it was found that over half of the tweets (52%) were related to health topics, and almost one-third of these tweets (30%) were posted by users who were not associated with a health profession, organization, or mission. The study also revealed that 17% of health tweets from providers and 74% from medical students included personal content against professional guidelines discouraging posting personal content [24]. These findings highlight concerns about potential conflicts of interest on Twitter as there is a limited or lack of medium to disclose such interest. These results illustrate the wide range of non–health-related users engaging in information sharing and health messaging on Twitter. To this end, we were mindful of not taking the role of primary care providers to provide medical advice via posts. We chose to offer evidence-based information and data so people can advocate for issues they care about or take questions to discuss with health care providers to make informed decisions. Using plain and simple language, devoid of medical jargon, may significantly help in engaging a diverse audience of nonhealth Twitter users.

### Limitations

There are a few limitations to note. The engagement data we used for this paper were generated directly from Twitter Analytics, which are provided free to all accounts. One of the major limitations of Twitter Analytics is the inability to know which exact accounts are viewing and engaging with our content. Twitter does not provide a list of accounts that have viewed our content, only the total number of views. Therefore, we did not have the ability to determine how many community members, community partners, or related professionals were viewing our content, nor did we know if our content, particularly our health literacy–related content, was reaching the communities for whom it was intended. Additionally, Twitter’s algorithm by which some posts are given priority to appear on other users’ content feeds is continuously changing and may also be driven by different levels of services. COMPASS has not yet decided to purchase a Twitter Blue subscription, which would contribute to prioritizing COMPASS-verified content across Twitter. We cannot currently comment or assess the effect of subscribing to premium Twitter services or using paid promotional tweets on impressions and engagement rates. Similarly, this paper covers a relatively short period (1 year) of Twitter Analytics. More information regarding the long-term use of Twitter as a vehicle for health advocacy is needed to better understand the use of Twitter as a tool for sustainable health advocacy promotion. Finally, the health literacy level of the web-based community who viewed our tweets was not accessed. Though our tweets have a great strength in simplicity and exclusion of medical jargons, the use of statistics and some health facts could have impaired the reading or understanding ability of the digital community of our post.

### Conclusions

Twitter has been useful in promoting health advocacy content, and our social media team aims to tap into and use other social media platforms such as Instagram, TikTok, and Facebook to reach several populations that may not use Twitter. In addition, while there is continuous improvement in our engagement with faculty, students, health practitioners, and community organizations, the next step is to strategize how social media can assist in increasing health awareness and mobilizing advocates and decision makers for policy change. O’Connor [25] emphasizes the use of IT, including social media, as a means to facilitate nurses (and health care enthusiasts) in contributing to policy design by facilitating networking and communication with senior leaders and legislators. As our social media–based health advocacy continues and expands onto new platforms, we anticipate that there will be ongoing changes in strategies, techniques, and styles to engage the audience. It will be resourceful to analyze the reactions we receive from the audience on these platforms as compared to Twitter.

## Abbreviations

COMPASS: Center for Community Programs, Innovation and Scholarship

## Data Availability

Twitter Analytics data will be made available from the corresponding author on reasonable request.
